# Spatial distribution and sociodemographic profile of transgender women and *travestis* undergoing rapid testing for syphilis and HIV in Manaus, Brazil, 2020-2021

**DOI:** 10.1590/S2237-96222024v33e2024388.especial.en

**Published:** 2024-01-10

**Authors:** Katia Cristina Bassichetto, Paula Andrea Morelli Fonseca, Rita Suely Bacuri de Queiroz, Rubens Kon, Maria Amélia de Sousa Mascena Veras

**Affiliations:** 1 Faculdade de Ciências Médicas da Santa Casa de São Paulo, São Paulo, SP, Brazil; 2 Instituto Leônidas e Maria Deane, Fiocruz Amazônia, Manaus, AM, Brazil; 3 Universidade de São Paulo, Faculdade de Medicina, São Paulo, SP, Brazil

**Keywords:** Personas Transgénero, Infecciones por VIH, Sífilis, Distribución Espacial, Estudios Transversales, Transgender People, HIV Infections, Syphilis, Spatial Distribution, Cross-Sectional Studies

## Abstract

**Objective:**

To analyze the spatial distribution of transgender women and *travestis* (TWTs), taking into consideration social vulnerability indicators and the results of rapid syphilis and HIV tests, in Manaus, the capital city of Amazonas state.

**Methods:**

A sampling method for hard-to-reach populations was used. Addresses were categorized according to the social vulnerability index . A total of 339 TWTs were recruited between 2020 and 2021, with 309 having their addresses mapped.

**Results:**

The majority (43.4%) lived in peripheral areas, with higher social vulnerability. Place of residence classification was not associated with syphilis (p-value = 0.578) and HIV (p-value = 0.885) positivity rates. Characteristics associated with higher seropositivity for sexually transmitted infections (STIs) included being aged 38 to 58 years, having completed high school, and history of sex work.

**Conclusion:**

A higher spatial concentration of TWTs was observed in areas with greater social vulnerability, although place of residence was not associated with the positivity rates for the STIs analyzed.

## Study contributions

**Main results:**

The majority of participants lived in areas with higher level of social vulnerability. The prevalence of syphilis and HIV was high, especially among older people, those with lower level of education and those with a history of engaging in sex work, with no association with place of residence.

**Implications for services:**

It is imperative to diversify strategies aimed at reaching marginalized and less visible groups, particularly in areas with greater social vulnerability. Furthermore, prevention efforts, as well as linkage and retention initiatives within programs targeting these health issues and promoting self-care, require reevaluation and optimization.

**Perspectives:**

Further research is necessary to deepen the understanding of the mechanisms driving exclusionary and inequity-producing dynamics faced by this population. Such studies could inform inclusive policies and contribute to reducing the prevalence of HIV and syphilis.

## RESUMEN

**Objetivo:**

Analizar la distribución espacial de mujeres trans y travestis (MTTr), considerando indicadores de vulnerabilidad social y resultados de pruebas rápidas de sífilis y VIH en Manaos, Brasil.

**Métodos:**

Se utilizó una técnica de muestreo para poblaciones de difícil acceso. Las direcciones residenciales se categorizaron según un índice de vulnerabilidad social. Se reclutaron 339 MTTr entre 2020 y 2021, de las cuales se mapearon las direcciones de 309.

**Resultados:**

La mayoría (43,4%) residía en áreas periféricas con mayor vulnerabilidad social. La clasificación del lugar de residencia no se asoció con las tasas de positividad para sífilis (p-valor = 0,578) ni VIH (p-valor = 0,885). Las características asociadas con una mayor seropositividad para infecciones de transmisión sexual (ITS) incluyeron edades entre 38 y 58 años, educación secundaria completa y antecedentes de trabajo sexual.

**Conclusión:**

Se observó una mayor concentración espacial de MTTr en áreas con mayor vulnerabilidad social, aunque el lugar de residencia no estuvo asociado con las tasas de positividad para las ITS analizadas.

## INTRODUCTION 

Syphilis and HIV remain significant public health challenges in various countries, profoundly impacting population health and well-being. Global estimates indicate that, in 2020, approximately 7.1 million people aged 15 to 49 acquired syphilis, while in 2022, 39 million people were living with HIV/AIDS by 2022.^1,2^

In Brazil, in 2022, the number of acquired syphilis cases was around 213,000, with a detection rate (DR) of 117.1 per 100,000 inhabitants; and, for people living with HIV/AIDS, (PLWHA) more than 1.1 million (17.1/100,000 inhabitants). Especially for the state of Amazonas, located in the Northern region of the country, over 5,000 cases of syphilis were reported, representing a DR of 99.2/100,000 inhabitants and 24,000 (2.3/100,000 inhabitants) of PLWHA.^3,4^ In its capital, Manaus, the DR for syphilis was 172.9/100,000 inhabitants,^5^ while for PLWHA, it was 54.1/100,000 inhabitants, ranking as the second capital city with the highest values for this indicator.^3^

For populations whose gender identity differs from the sex assigned at birth,^6^ there are currently no official estimates for DR of sexually transmitted infections (STIs). In order to address this gap, prevalence studies on various STIs have been conducted, showing that this population group, especially trans women and *travestis* (TWTs), is disproportionately affected by STIs in several countries, including Brazil, where this variable is not included in the notification forms for syphilis and HIV of the Notifiable Health Conditions Information System.^7-9^ This has been explained by several factors, including engagement in high-risk sexual behaviors, multiple sexual partners, non-use of condoms during receptive anal sex, and the use of psychoactive substances. Combined with the anticipated fear of or actual experiences with stigma and discrimination, these factors create significant barriers to accessing healthcare services, exacerbating the high prevalence of STIs in this population.^10-14^

Despite the increase in the number of studies on sexual and gender diversity, few studies have focused on measuring inequalities in social determinants of health among the trans population.^15^ In order to describe how environmental and socioeconomic contexts influences health outcomes, socio-spatial analysis has been used in epidemiological studies, considering living conditions and territories of residence. Identifying potential patterns can contribute to the assessment of the health profile, including morbidity and associated factors, as well as the resources used by people to overcome barriers and access healthcare. It can also improve the design of interventions tailored to health needs of these population groups, and, consequently, enhance their quality of life.

In this context, this study aims to analyze the spatial distribution of transgender women and *travestis* recruited in a multicenter study, considering the areas according to social vulnerability indicators and results of rapid tests (RTs) for syphilis and HIV in Manaus, Amazonas state.

## METHODS

### Study design

This was an observational study conducted with transgender women and *travestis* in Manaus, Amazonas state, between 2020 and 2021.

### Setting

Data were collected from the TransOdara cross-sectional study – *Study on the prevalence of syphilis and other sexually transmitted infections among transgender women and travestis in Brazil: care and prevention*, which involved TWTs aged 18 years and older, in five Brazilian capitals (São Paulo, Campo Grande, Manaus, Porto Alegre and Salvador), between 2019 and 2021, were used. A mixed methods approach – quantitative and qualitative – was used, under the coordination of the Faculdade de Ciências Médicas da Santa Casa de São Paulo and the Instituto de Saúde Coletiva da Universidade Federal da Bahia. The original project aimed to estimate the prevalence of syphilis, HIV, *Neisseria gonorrhoeae*, *Chlamydia trachomatis*, Human papillomavirus and hepatitis A, B and C viruses, as well as to explore participants’ perceptions of syphilis infection.

Given that this is a hard-to-reach population, living in marginalized conditions and sparsely distributed across urban areas, an appropriate sampling method was used – respondent-driven sampling (RDS) –, aimed to overcome the limitations of traditional probabilistic methods for populations with unknown size. This technique is preferred over convenience sampling.^16^

The recruitment of the study population began by selecting “seeds” who were able to identify potential participants within their social networks. For this purpose, social spaces and institutions providing services to trans people were visited.^17^ Recruitment was carried out in waves, with the first wave consisting of participants referred by the seeds, the second wave composed of participants referred by those in the first wave, and so forth, until the projected sample size was reached. In order to avoid excessive concentration of participants from some social networks, a maximum number of invitations for each seed was limited.

### Study size

The sample size for each site was calculated based on the prevalence of active syphilis, defined by titers greater than 1:8 of the venereal disease research laboratory. The results of the Divas study were used to estimate syphilis prevalence for each site.^18^ The standard error was calculated using the method proposed by Salganik,^19^ which does not assume a known population size.

The multicenter project recruited 1,317 people, for a calculated sample of 1,250 people. A baseline questionnaire was administered and tests to detect syphilis and HIV, among other STIs, were offered to all participants. The study protocol also included medical consultations and prescribed treatment for any conditions identified through testing and clinical evaluations.

### Participants

**Figure 1:**
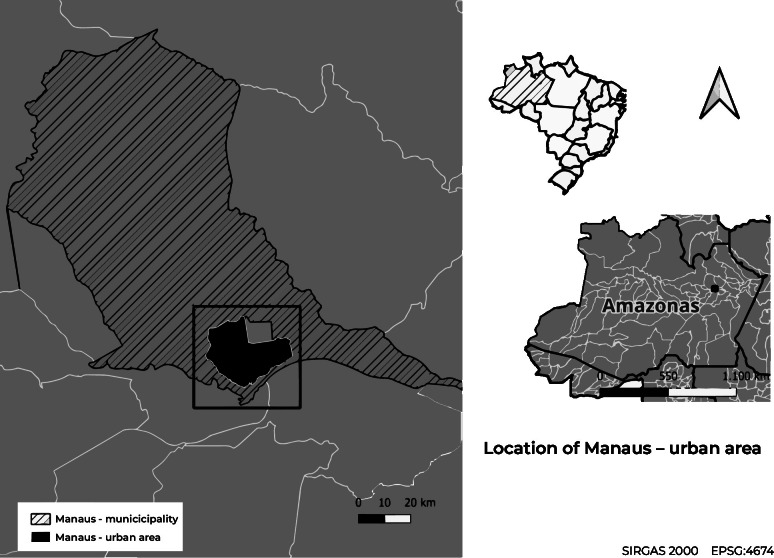
Map showing the location of the municipality and urban area of Manaus, Brazil

This study used data from participants living in the metropolitan region of Manaus (Figure 1), between November 2020 and April 2021.

### Variables, data sources and measurement

During the original study, all participants underwent RTs for syphilis, and all but one, were tested for HIV. The results of the RTs performed were obtained from the Clinical Assessment and Follow-up Form used in the research protocol. Those characterized as living in street situations were considered as a specific subgroup in the analysis. Those with incomplete or insufficient address information for precise geolocation were excluded.

For geoprocessing, the addresses provided by the participants were reviewed for consistency, correlating data on street names, neighborhoods and postal codes, making corrections when needed. Subsequently, the addresses were geolocated using the BatchGeo application (available at www.batchgeo.com*),* which generated a keyhole markup language format file. Then, the geographic information software QGIS (QGIS – GIS software –, version 3.24, QGIS Geographic Information System. Open-Source Geospatial Foundation Project. http://qgis.osgeo.org, 2022) was used to check and adjust geocoding results, integrating them with other geographic layers of interest, particularly, the Human Development Units (HDUs), obtained from the website of the Atlas of Social Development by the Institute for Applied Economic Research (*Instituto de Pesquisa Econômica Aplicada* - IPEA).

The 198 HDUs in Manaus were categorized according to the Social Vulnerability Index (SVI), developed by IPEA. This index, constructed from sixteen indicators from the Social Vulnerability block of the Atlas of Human Development in Brazil, synthesizes three dimensions: urban infrastructure, human capital/income, and labor. The data source was the census tracts from the 2010 Brazilian Census, and the index is available for all municipalities and HDUs in major metropolitan areas. The SVI aims to signal access and the absence/insufficiency of certain “assets” in areas of Brazilian territory, which “should be available to every citizen, by force of State action”.^20^

The SVI value ranges from 0 to 1, and the higher the value, the greater the vulnerability of the municipality or HDU. Originally, the index was categorized as very low (0 to 0.200), low (0.201 to 0.300), medium (0.301 to 0.400), high (0.401 to 0.500) and very high (0.501 to 1.000) vulnerability. For this study, three categories for analysis were used: very low/low (0 to 0.300), medium (0.301 to 0.400) and high/very high (0.401 to 1.000). Participants were distributed according to the three categories of social vulnerability, considering the reactive results of the RTs for syphilis, HIV and coinfection (syphilis and HIV).

The variables selected to characterize the group were obtained from the questionnaires administered during the TransOdara study: age, disaggregated into age groups (18-29; 30-58 years); race/skin color (Black/mixed-race; White/Asian/Indigenous); education level (elementary school; high school); income (up to a minimum wage; greater than a minimum wage), sex in exchange for money or other goods (yes; no); and living in street situations (yes; no).

### Statistical methods

For statistical decision making, Fisher’s exact test was performed to assess the significance of the association of each condition separately – syphilis, HIV and coinfection, relative to the overall participant group, considering a p-value <0.05 and a 95% confidence interval. The Stata, version 14.2, was used.

### Ethical aspects

This project was approved by the Ethics and Research Committee of Santa Casa de Misericórdia de São Paulo, under opinion No. 3,126,815, on January 30, 2019 (Certificate of Presentation for Ethical Appraisal No. 05585518.7.0000.5479). Only participants who signed the Free and Informed Consent Form were included in the study, and all. participants retained the right to withdraw at any time without affecting their continued care in health services.

## RESULTS

Of the 339 study participants recruited for the study in Manaus, 16 participants were excluded from this analysis (15 due to untraceable addresses and one residing in Minas Gerais state). Among the remaining 323 participants, 14 were living in street situations. Thus, 309 (91.1%) had their addresses mapped.

All 323 participants analyzed underwent RT for syphilis (196, or 60.6%, with reactive results; 127, or 39.4%, non-reactive results. Among 322 participants tested for HIV, 116 (36.0%) had reactive results, while 206 (64.0%) were non-reactive. When considering results for both tests simultaneously, 89 participants (27.6%) showed reactive results for both, while 100 participants (31.0%) were non-reactive for both tests.

**Figure 2:**
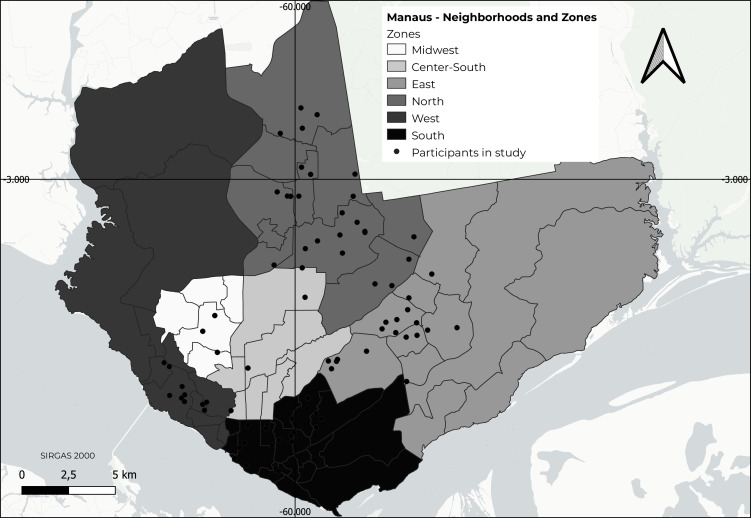
Spatial distribution of participants in the TransOdara study, according to Zones and Neighborhoods, Manaus, Brazil, 2020-2021

Figure 2 shows the spatial distribution of the study participants whose addresses were located, with the general population of each zone being used as a reference to characterize this distribution. Manaus is divided into six administrative zones: Midwest, Center-South, East, North, West and South; among these, the North and East zones are the most densely populated. A territorial dispersion was observed across the city, with a lower concentration in the Center-South zone (n=14; 9.98/100 thousand inhabitants) and a higher concentration in the South zone (n=89; 25.21/100 thousand inhabitants).

Regarding socioeconomic and demographic characteristics, participants were evenly distribution across the age groups of 18 to 29 years (49.9%) and 30 to 58 years (50.1%); the majority self-identified as Black/mixed-race (76.3%); had at least a high school education (77.9%); reported an income of up to one minimum wage (68.4%); and who had already had sex in exchange for money, drugs, housing or other goods (66.4%) (Table 1).

**Table 1:** Distribution (%) of participants in the TransOdara Project, according to socioeconomic and demographic variables and rapid test (RT) results for syphilis and HIV, Manaus, Brazil, 2020-2021 (n=309)

Variables	RT reactive for syphilis		RT reactive for HIV		RT reactive for syphilis and HIV		Total
	n (%)	p-value	n (%)	p-value	n (%)	p-value	n (%)
**Age group (years)**		<0.001			<0.001		<0.001
18-29	81 (41.3)		36 (31.0)		26 (29.2)		161 (49.9)
30-58	115 (58.7)		80 (69.0)		63 (70.8)		162 (50.1)
**Race/skin color**		0.5335			0.892		0.884
Black/mixed-race	149 (76.4)		88 (75.9)		69 (77.5)		245 (76.3)
White/Asian/Indigenous	46 (23.6)		28 (24.1)		20 (22.5)		76 (23.7)
**Education level**		0.006			0.017		0.025
Elementary education	53 (27.2)		34 (29.6)		27 (30.7)		71 (22.1)
High school	142 (72.8)		81 (70.4)		61 (69.3)		251 (77.9)
**Income (minimum wage)**		0.7133			0.385		0.287
≤ 1	136 (69.4)		83 (71.6)		65 (73.0)		221 (68.4)
>1	60 (30.6)		33 (28.4)		24 (27.0)		102 (31.6)
**Sex in exchange for money or other goods**		<0.001			0.039		0.018
Yes	118 (75.2)		67 (75.3)		54 (78.3)		178 (66.4)
No	39 (24.8)		22 (24.7)		15 (21.7)		90 (33.6)
**Living in street situations**		0.263			0.610		0.931
Yes	11 (5.6)		5 (4.3)		4 (4.5)		14 (4.3)
No	185 (94.4)		111 (95.7)		85 (95.5)		309 (95.7)

As for the distribution of these characteristics according to subgroups, it could be seen that, participants with reactive syphilis tests showed significantly higher proportions of older age (58.7%; p-value <0.001), lower education level (27.2%; p-value = 0.006) and a history of having sex in exchange for money or other goods (75.2%; p-value <0.001). These same variables were significantly associated with the subgroup with reactive tests for HIV, with higher proportions observed for older age (69.0%; p-value <0.001), lower education level (29.6%; p-value = 0.048) and who reported experience in exchanging sex for money or other goods (75.3%; p-value = 0.030). The same occurred in the subgroup with reactive tests for both conditions simultaneously – older age group (70.8%; p-value <0.001), lower education level (30.7%; p-value = 0.022) and those who reported experience of exchanging sex for money or other goods (78.3%; p-value = 0.016). No significant difference was observed in any of the groups regarding race/skin color and income. Living in street situations was also not associated with seropositivity for these STIs (Table 1).

**Figure 3:**
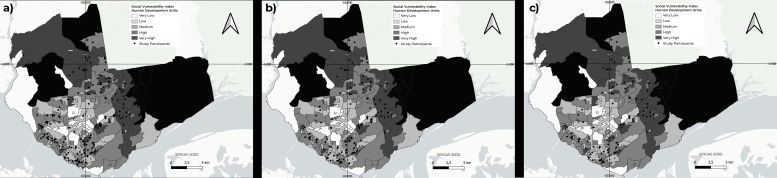
Spatial distribution of participants in the TransOdara Project, with positive rapid test (RT) results for syphilis (a), HIV (b) and both (c), according to areas of social vulnerability, Manaus, Brazil, 2020-2021

When analyzing the distribution of the total number of participants according to the SVI, the majority (n=140; 43.4%) lived in areas of high/very high vulnerability, 115 (35.6%) in areas of medium vulnerability and 54 (16.7%) in areas of low/very low vulnerability, in addition to 14 (4.3%) who lived in street situations. The distribution of participants with reactive tests for syphilis, HIV and both, according to areas of social vulnerability, was similar to that observed among participants with non-reactive RTs, with no statistically significant difference detected between areas (Table 2 and Figure 3).

**Table 2:** Distribution (%) and 95% confidence intervals (95%CI) of the participants in the TransOdara Project, according to areas of social vulnerability and rapid test (RT) results for syphilis and HIV, Manaus, Brazil, 2020-2021 (n=323)

	RT reactive for syphilis			RT reactive for HIV			**RT reactive for syphilis and HIV**			**Total**	
	n	% (95%CI)	p-value	n	% (95%CI)	p-value	n	% (95%CI)	p-value	n	% (95%CI)
**Social vulnerability – area**			**0.578**			**0.885**			**0.881**		
High/very high	84	42.9 (35.8;50.1)		47	40.5 (31.5;50.0)		36	40.4 (30.2;51.4)		140	43.4 (37.9;48.9)
Medium	69	35.2 (28.5;42.3)		43	37.1 (28.3;46.5)		32	36.0 (26.1;46.8)		115	35.6 (30.4;41.1)
Low/very low	32	16.3 (11.4;22.3)		21	18.1 (11.6;26.3)		17	19.1 (11.5;28.8)		54	16.7 (12.8;21.2)
Living in street situations	11	5.6 (2.8;9.8)		5	4.3 (1.4;9.8)		4	4.5 (1.2;11.1)		14	4.3 (2.4;7.2)

## DISCUSSION 

This study revealed that the majority of participants lived in areas classified as having *high/very high* and *medium* social vulnerability, where there is still a lack of service provision. Together, these areas account for approximately 70% of the population of Manaus. The spatial distribution of study participants, when referenced to the population of each zone, showed a higher concentration in the South zone and lower representation in the Center-South zone, with other zones presenting distributions close to the city average.

The Center-South zone is characterized by an upper-middle-class population with high purchasing power, and high housing costs. The South zone encompasses the city’s historic and commercial center, health services, public policy support centers and social assistance services, aimed at the LGBTQIAP+ population. The offer of specialized services may explain the choice of this area as a place of residence. This region is marked by strong social and architectural contrasts, including numerous precarious housing units, located in vulnerable areas, such as valley bottoms and creek banks, narrow and shallow waterways. Additionally, the South zone is known as an established center for prostitution, making it more attractive to transgender women facing socioeconomic vulnerability.^20,21^

Place of residence was not associated with syphilis and HIV positivity rates among the study population, although the participants’ addresses were distributed across all areas analyzed. This result is similar to that of another study conducted in São Paulo, capital city of São Paulo state.^22^

The relationship between age group (the study participants being older) and outcomes may be explained by longer exposure to these STIs. This finding differs from that of a systematic review of transgender populations, taking into consideration the results of 25 studies conducted in 11 countries, including Brazil, which reported higher prevalence of HIV among younger people.^23^

Low educational level was also associated with the outcomes, a classical indicator of social vulnerability, not only among the trans population, but also in other studies involving diverse populations.^24^ However, for TWTs, this condition may be related to high school dropout rates, due to lack of family support, experiences of transphobia and discrimination.^13,24^

This factor can limit access to the essential health information required to adopt self-care strategies. In addition, it can hinder access to employment and income, and, consequently, sex work may become a means of survival, exposing these individuals to risks associated with this type of work, such as having multiple partners and engaging in unprotected sexual practices.^14,25^

The proportion of the trans population with up to elementary education in this study (22%) (TransOdara - Manaus) was similar to that observed in TransOdara – municipality of São Paulo (MSP) (24%),^23^ but lower than that observed in a mapping of the trans population conducted in the same municipality (42%).^26^ Recruitment strategies may explain the differences found in the educational profile among the trans population across studies.

The association between the positivity rate of the outcomes and a history of sex work in this study (66.4%) was higher than that indicated in a systematic review conducted in the United States (37.9%); lower than that observed in a cohort study with TWTs, conducted in the city of Rio de Janeiro (78.6%);^27^ and similar to that observed among the TransOdara participants, recruited in the MSP (68.8%).^22^

It is worth mentioning that, although no statistical association was found between living in street situations at the time of participation in the project and the reactive RT results for the outcomes analyzed, it is known that this condition exacerbates the vulnerability of an already marginalized group facing numerous challenges.^28^ It is possible that the low number of participants in this condition did not allow this association to be evidenced. Alternatively, the risk might be more closely linked to other socioenvironmental, socioeconomic, and behavioral conditions or the transient nature of homelessness itself.

Among the limitations of this study, we can highlight the recruitment method used (RDS), subject to the characteristics of the social networks of this population, which are poorly understood. Therefore, caution is recommended when interpreting the results. However, in the absence of better strategies, RDS remains a valuable method for studying hard-to-reach populations like TWTs in various contexts.^16^ Another limitation was the occurrence of the COVID-19 pandemic, concomitant with this study, since it influenced the recruitment chains and the logistics of the locations where the research was conducted, complicating efforts to address participants’ additional healthcare needs. Finally, although this study was not originally designed to analyze the socio-spatial distribution of the outcomes of interest, it contributed to showing that these population groups are mainly concentrated in territories of greater social vulnerability. It is also worth mentioning that, although the SVI was constructed based on data from the 2010 Census, and there may be some discrepancy in relation to the current situation, it remains the most available dataset. It is believed that relative vulnerability distribution has not undergone drastic changes.

It can be stated that the analysis employed added value by identifying priority locations for public policies aimed at promoting better living and health conditions for this population.^25,29^ The high rates of syphilis and HIV positivity in the population of this study and the predominant distribution in areas of high and very high social vulnerability underscore the need to implement prevention, linkage, and retention programs for prophylaxis and recommended treatments within these territories.^30^ This requires the diversification of strategies to expand the reach of invisible key populations, such as TWTs, and, consequently , increase their adherence to health services.

In conclusion, this study contributed to characterizing the distribution of the TWTs participating in the study across the city’s territories, in addition to identifying a subgroup with heightened vulnerabilities, although this was not explicitly reflected in the spatial distribution.

By expanding the understanding of the mechanisms driving exclusion and inequities affecting this population, this study aims to inspire healthcare professionals, policymakers, and civil society to address and reverse these realities.
