# Supplementary material for: Spatial distribution and sociodemographic profile of transgender women and travestis undergoing rapid testing for syphilis and HIV in Manaus, Brazil, 2020-2021
Source: Epidemiol Serv Saude. 2024 Jan 10;33(spe1):e2024388. doi: 10.1590/S2237-96222024v33e2024388.especial.en (PMC11734660; doi:10.1590/S2237-96222024v33e2024388.especial.en)
Supplement: Translation

# Translation: Distribuição espacial e perfil sociodemográfico de mulheres transexuais e travestis com teste rápido para sífilis e HIV em Manaus, 2020-2021

**DOI:** 10.1590/S2237-96222024v33e2024388.especial.pt

**Authors:** Katia Cristina Bassichetto, Paula Andrea Morelli Fonseca, Rita Suely Bacuri de Queiroz, Rubens Kon, Maria Amélia de Sousa Mascena Veras

**Affiliations:** 1 Faculdade de Ciências Médicas da Santa Casa de São Paulo, São Paulo, SP, Brasil; 2 Instituto Leônidas e Maria Deane, Fiocruz Amazônia, Manaus, AM, Brasil; 3 Universidade de São Paulo, Faculdade de Medicina, São Paulo, SP, Brasil

**Keywords:** Pessoas Transgênero, Infecções por HIV, Sífilis, Distribuição espacial, Estudos Transversais

## Abstract

**Objetivo:**

Analisar a distribuição espacial de mulheres trans e travestis (MTTr), considerando-se indicadores de vulnerabilidade social e resultados de testes rápidos de sífilis e HIV, em Manaus-AM.

**Métodos:**

Utilizou-se técnica de amostragem para populações de difícil acesso. Os endereços foram categorizados segundo índice de vulnerabilidade social. Foram recrutadas 339 MTTr entre 2020 e 2021, e 309 tiveram seus endereços mapeados.

**Resultados:**

A maioria (43,4%) morava em áreas periféricas, com maior vulnerabilidade social. A classificação do local de moradia não se mostrou associada às taxas de positividade de sífilis (p-valor = 0,578) e HIV (p-valor = 0,885). As características associadas à maior soropositividade para as infecções sexualmente transmissíveis (IST) foram idade entre 38 e 58 anos, ensino médio completo e histórico de trabalho sexual.

**Conclusão:**

Observou-se maior concentração espacial de MTTr em áreas de maior vulnerabilidade social, embora o local de moradia não tenha se mostrado associado às taxas de positividade para as IST analisadas.

## Contribuições do estudo

**Principais resultados:**

A maioria das participantes residia em áreas de maior vulnerabilidade social. Foi alta a detecção de sífilis e HIV, principalmente entre mais velhas, menos escolarizadas e com histórico de trabalho sexual, sem associação com o local de moradia.

**Implicações para os serviços:**

Há necessidade de diversificar estratégias para alcançar grupos invisibilizados, considerando-se as áreas de maior vulnerabilidade, e rever ações de prevenção, vinculação e retenção aos programas dirigidos a estes agravos e ao autocuidado.

**Perspectivas:**

Investigações complementares que ampliem a compreensão dos mecanismos que levam à dinâmica excludente e produtora de desigualdade a que esta população está submetida podem subsidiar políticas de inclusão e redução das prevalências de HIV e sífilis.

## INTRODUÇÃO 

A sífilis e o HIV continuam a representar desafios significativos para a saúde pública em diversos países, tendo impacto profundo na saúde e bem-estar das populações. Há estimativas globais de que, em 2020, aproximadamente 7,1 milhões de pessoas, entre 15 e 49 anos, adquiriram sífilis e que, em 2022, 39 milhões estavam vivendo com HIV/aids.^1,2^

No Brasil, em 2022, o número de casos de sífilis adquirida foi cerca de 213 mil, com taxa de detecção (TD) de 117,1 por 100 mil habitantes; e, para pessoas vivendo com o HIV/aids, (PVHA) mais de 1,1 milhão (17,1/100 mil habitantes). Especialmente para o estado do Amazonas, região Norte do país, foram notificados mais de 5 mil casos de sífilis, representando uma TD de 99,2/100 mil habitantes e 24 mil (2,3/100 mil habitantes) de PVHA.^3,4^ Em sua capital, Manaus, a TD para sífilis foi de 172,9/100 mil habitantes,^5^ e para PVHA, de 54,1/100 mil habitantes, ocupando o 2º lugar no *ranking* das capitais com maiores valores deste indicador.^3^

Para populações que apresentam uma identidade de gênero diferente do sexo atribuído ao nascimento,^6^ não há, atualmente, estimativas oficiais da TD de infecções sexualmente transmissíveis (IST). Para suprir essa lacuna, estudos de prevalência de diversas IST têm sido desenvolvidos, mostrando que esse grupo populacional, em especial mulheres trans e travestis (MTTr), é desproporcionalmente afetado pelas IST em diversos países, incluindo o Brasil, onde esta variável não consta nas fichas de notificação de sífilis e HIV do Sistema de Informação de Agravos de Notificação.^7-9^ Isso tem sido explicado por vários fatores, como a adoção de práticas sexuais de risco, parcerias múltiplas, não utilização de preservativo durante sexo anal receptivo e uso de substâncias psicoativas, que, combinados ao medo antecipado de sofrerem ou mesmo vivenciarem estigma e discriminação, se configuram como dificuldades abrangentes de acesso aos serviços de saúde e agravam o cenário de altas prevalências de IST nessa população.^10-14^

Apesar do aumento de estudos envolvendo diversidade sexual e de gênero, poucos se dedicam a mensurar desigualdades nos determinantes sociais de saúde na população trans.^15^ Para buscar descrever os modos de interferência do contexto ambiental e socioeconômico nos desfechos de saúde, tem-se utilizado análise socioespacial em estudos epidemiológicos, considerando-se condições de vida e territórios de residência. A verificação de possíveis padrões pode contribuir para a avaliação do perfil de saúde, incluindo morbidade e fatores associados, bem como recursos utilizados pelas pessoas para vencer as dificuldades vivenciadas e obter atenção à saúde. Pode, ainda, aprimorar o delineamento de intervenções, de forma que sejam mais focadas nas necessidades de saúde desses grupos populacionais, e, por conseguinte, possam melhorar a sua qualidade de vida. 

Neste sentido, este estudo tem como objetivo analisar a distribuição espacial de mulheres trans e travestis, recrutadas em um estudo multicêntrico, considerando as áreas segundo indicadores de vulnerabilidade social e resultados de testes rápidos (TRs) de sífilis e HIV em Manaus.

## MÉTODOS

### Delineamento

Trata-se de um estudo observacional realizado com mulheres trans e travestis em Manaus, nos anos de 2020 e 2021.

### Contexto

Foram utilizados dados coletados no TransOdara, de natureza transversal – *Estudo de prevalência da sífilis e outras infecções sexualmente transmissíveis entre mulheres transexuais e travestis no Brasil: cuidado e prevenção*, que envolveu MTTr, maiores de 18 anos, em cinco capitais brasileiras (São Paulo, Campo Grande, Manaus, Porto Alegre e Salvador), entre 2019 e 2021. Foi utilizada abordagem mista – quantitativa e qualitativa –, sob coordenação da Faculdade de Ciências Médicas da Santa Casa de São Paulo e do Instituto de Saúde Coletiva da Universidade Federal da Bahia. O projeto original estimou a prevalência de sífilis, HIV, *Neisseria gonorrhoeae*, *Chlamydia trachomatis*, Papiloma vírus humano e os vírus das hepatites A, B e C, além de reconhecer os significados atribuídos à infecção por sífilis, entre as participantes. 

Considerando tratar-se de população de difícil acesso, vivendo em condições de marginalização e esparsamente distribuída nas áreas urbanas, foi utilizada técnica de amostragem apropriada – *respondent-driven sampling* (RDS) –, que busca superar as limitações da amostragem probabilística clássica para este tipo de população, cujo tamanho é usualmente desconhecido. Esta técnica tem sido considerada preferível aos métodos de amostragem por conveniência.^16^

O recrutamento da população do estudo foi iniciado pela seleção de “sementes”, capazes de identificar possíveis participantes em suas redes sociais. Para isto, foram visitados espaços de sociabilização e instituições com serviços para pessoas trans.^17^ O recrutamento se expandiu em ondas, sendo a primeira formada pelas participantes indicadas pelas sementes, a segunda, pelas participantes indicadas pelas componentes da primeira onda, e assim sucessivamente, até que fosse atingida a quantidade projetada para a amostra. Para evitar a concentração excessiva de participantes de algumas redes sociais, limitou-se o número máximo de convites para cada semente. 

### Tamanho do estudo

O cálculo amostral de cada sítio foi estimado pela prevalência de sífilis ativa, considerando títulos maiores que 1:8 do *venereal disease research laboratory*. Os resultados do estudo Divas foram utilizados para o cálculo das estimativas de prevalência de sífilis em cada sítio.^18^ O erro-padrão foi calculado usando-se o método proposto por Salganik,^19^ que não assume um tamanho de população conhecido.

O projeto multicêntrico recrutou 1.317 pessoas, para uma amostra calculada de 1.250 pessoas. Foi aplicado questionário *baseline* e oferecidos testes para detecção de sífilis e HIV, entre outras IST, a todas as participantes. O fluxo da pesquisa também previa que passassem por consultas médicas e tivessem tratamento prescrito para as condições eventualmente identificadas nos testes e na avaliação clínica. 

### Participantes

Este estudo utilizou dados das participantes moradoras na região metropolitana de Manaus (Figura 1), entre novembro de 2020 a abril de 2021. 

**Figura 1:** Mapa de localização do município e área urbana de Manaus

### Variáveis, fontes de dados e mensuração

Durante o estudo original, todas realizaram TR para sífilis e, com exceção de uma, para HIV. Os resultados dos TRs realizados foram obtidos do Formulário de Avaliação Clínica e Seguimento, aplicado no fluxo da referida pesquisa. Aquelas caracterizadas como estando em situação de rua foram consideradas como um subgrupo específico na análise. Foram excluídas aquelas com endereços incompletos ou insuficientes para uma localização precisa. 

Para o geoprocessamento, os endereços informados pelas participantes foram analisados em termos de sua consistência, correlacionando os dados de logradouro, bairro e Código de Endereçamento Postal, procedendo a correções quando indicado. Após esta etapa, os endereços foram geolocalizados, utilizando-se o aplicativo Batchgeo (disponível *online* em www.batchgeo.com), que gerou um arquivo em formato *keyhole markup language*. Na sequência, foi utilizado o *software* de informação geográfica QGIS (QGIS – *software* GIS –, versão 3.24, QGIS Geographic Information System. Open-Source Geospatial Foundation Project. http://qgis.osgeo.org, 2022) para conferência e ajuste dos resultados da geocodificação e processamento conjunto com outras camadas geográficas de interesse, em particular o das Unidades de Desenvolvimento Humano (UDH), obtido no *site* do Atlas de Desenvolvimento Social do Instituto de Pesquisa Econômica Aplicada (Ipea). 

As 198 UDH de Manaus foram categorizadas, segundo o Índice de Vulnerabilidade Social (IVS), adotado pelo Ipea. Este índice, construído a partir de dezesseis indicadores do bloco de Vulnerabilidade Social do Atlas do Desenvolvimento Humano no Brasil, sintetiza três dimensões – infraestrutura urbana, capital humano/renda e trabalho. A fonte dos dados é constituída dos setores censitários do Censo Demográfico de 2010 do Instituto Brasileiro de Geografia e Estatística, e está disponível para todos os municípios do país e para as UDH das principais regiões metropolitanas. O IVS se propõe a sinalizar o acesso e a ausência/insuficiência de alguns “ativos” em áreas do território brasileiro, que “deveriam estar à disposição de todo cidadão, por força da ação do Estado”.^20^

O valor do IVS varia entre 0 e 1, sendo que, quanto maior o valor, maior a vulnerabilidade do município ou UDH. Originalmente, o índice foi categorizado como vulnerabilidade muito baixa (0 a 0,200), baixa (0,201 a 0,300), média (0,301 a 0,400), alta (0,401 a 0,500) e muito alta (0,501 a 1,000). Neste trabalho, utilizamos três categorias para análise: muito baixa/baixa (0 a 0,300), média (0,301 a 0,400) e alta/muito alta (0,401 a 1,000). As participantes foram distribuídas segundo as três categorias de vulnerabilidade social, considerando-se os resultados reagentes dos TRs realizados para sífilis, HIV e coinfecção (sífilis e HIV). 

As variáveis selecionadas para caracterização do grupo foram obtidas dos questionários aplicados durante o estudo TransOdara: idade, desagregada em faixas etárias (18-29; 30-58 anos); raça/cor da pele (preta/parta; branca/amarela/indígena); escolaridade (ensino fundamental; ensino médio); renda (até um salário mínimo; maior que um salário-mínimo), sexo em troca de dinheiro ou outros bens (sim; não); e situação de rua (sim; não). 

### Métodos estatísticos

Para tomada de decisão estatística, foi realizado o teste exato de Fisher, a fim de se avaliar a significância da associação de cada condição em separado – sífilis, HIV e coinfecção, por referência ao conjunto das participantes, considerando-se valor de p-valor <0,05 e intervalo de confiança de 95%. Utilizou-se o *software* Stata, versão 14.2.

### Aspectos éticos

Este projeto foi aprovado pelo Comitê de Ética e Pesquisa da Santa Casa de Misericórdia de São Paulo, sob o parecer n^o^ 3.126.815, em 30 de janeiro de 2019 (Certificado de Apresentação para Apreciação Ética n^o^ 05585518.7.0000.5479). Somente as participantes que assinaram o Termo de Consentimento Livre e Esclarecido foram incluídas no estudo. Foi, também, assegurada a possibilidade de desistência da participação a qualquer momento, sem que isso interferisse no seguimento nas unidades de saúde.

## RESULTADOS

Das 339 participantes do estudo recrutadas em Manaus, foram excluídas desta análise 16 participantes (15 cujos endereços não foram localizados e uma residente em Minas Gerais). Das 323 restantes, 14 se encontravam em situação de rua. Desse modo, 309 (91,1%) tiveram seus endereços mapeados. 

Todas as 323 participantes analisadas realizaram TR para sífilis (196, ou 60,6%, com resultados reagentes; 127, ou 39,4%, não reagentes) e 322 para HIV (116, ou 36,0%, com resultados reagentes; 206, ou 64,0% não reagentes). Considerando-se os resultados para ambos os testes simultaneamente, 89 (27,6%) das participantes tiveram resultados reagentes, e 100 (31,0%), não reagentes.

A Figura 2 apresenta a distribuição espacial do conjunto das participantes do estudo que tiveram endereços localizados, sendo que a população geral de cada zona foi utilizada como referência para caracterização desta distribuição. Manaus está dividida em seis zonas administrativas: Centro-Oeste, Centro-Sul, Leste, Norte, Oeste e Sul; entre estas, as zonas Norte e Leste são as mais populosas. Observou-se uma dispersão territorial pela cidade, com menor concentração na região Centro-Sul (n=14; 9,98/100 mil habitantes) e maior concentração na zona Sul (n=89; 25,21/100 mil habitantes).

**Figura 2:** Distribuição espacial das participantes do estudo TransOdara, segundo Zonas e Bairros, Manaus, 2020-2021

Com relação às características socioeconômicas e demográficas, houve uma distribuição equivalente nas faixas etárias de 18 a 29 anos (49,9%) e 30 a 58 anos (50,1%); a maior parte se referiu como de raça/cor da pele preta/parda (76,3%); com escolaridade igual ou superior ao ensino médio (77,9%); com renda de até um salário mínimo (68,4%); e que já havia praticado sexo em troca de dinheiro, drogas, moradia ou outros bens (66,4%) (Tabela 1).

**Tabela 1:** Distribuição em (%) das participantes do Projeto TransOdara, segundo variáveis socioeconômicas e demográficas e resultado do teste rápido (TR) para sífilis e HIV, Manaus, 2020-2021 (n=309)

Variáveis	TR reagente para sífilis		TR reagente para HIV		TR reagente para sífilis e HIV		Total
	n (%)	p-valor	n (%)	p-valor	n (%)	p-valor	n (%)
**Faixa etária (anos)**	<0,001			<0,001		<0,001	
18-29	81 (41,3)		36 (31,0)		26 (29,2)		161 (49,9)
30-58	115 (58,7)		80 (69,0)		63 (70,8)		162 (50,1)
**Raça/cor da pele**	0,5335			0,892		0,884	
Preta/parda	149 (76,4)		88 (75,9)		69 (77,5)		245 (76,3)
Branca/amarela/indígena	46 (23,6)		28 (24,1)		20 (22,5)		76 (23,7)
**Escolaridade**	0,006			0,017		0,025	
Ensino fundamental	53 (27,2)		34 (29,6)		27 (30,7)		71 (22,1)
Ensino médio	142 (72,8)		81 (70,4)		61 (69,3)		251 (77,9)
**Renda (salário mínimo)**	0,7133			0,385		0,287	
≤1	136 (69,4)		83 (71,6)		65 (73,0)		221 (68,4)
>1	60 (30,6)		33 (28,4)		24 (27,0)		102 (31,6)
**Sexo em troca de dinheiro ou outros bens**		<0,001			0,039		0,018
Sim	118 (75,2)		67 (75,3)		54 (78,3)		178 (66,4)
Não	39 (24,8)		22 (24,7)		15 (21,7)		90 (33,6)
**Situação de rua**		0,263			0,610		0,931
Sim	11 (5,6)		5 (4,3)		4 (4,5)		14 (4,3)
Não	185 (94,4)		111 (95,7)		85 (95,5)		309 (95,7)

No que se refere à distribuição destas características segundo subgrupos, observa-se que, para aquele com testes reagentes para sífilis, houve uma proporção significativamente aumentada de pessoas de maior faixa etária (58,7%; p-valor <0,001), de menor escolaridade (27,2%; p-valor = 0,006) e que relataram experiência na prática de sexo em troca de dinheiro ou outros bens (75,2%; p-valor <0,001). Essas mesmas variáveis mostraram-se associadas ao subgrupo com testes reagentes para HIV, com proporção significativamente aumentada para aquelas de maior faixa etária (69,0%; p-valor <0,001), de menor escolaridade (29,6%; p-valor = 0,048) e que relataram experiência de trocar sexo por dinheiro ou outros bens (75,3%; p-valor = 0,030). O mesmo ocorreu no subgrupo com testes reagentes para as duas condições simultaneamente – de maior faixa etária (70,8%; p-valor <0,001), de menor escolaridade (30,7%; p- valor = 0,022) e que relataram experiência de trocar sexo por dinheiro ou outros bens (78,3%; p-valor = 0,016). Não se observou diferença significativa, para nenhum dos grupos, em relação à raça/cor da pele e renda. Estar em situação de rua também não se mostrou associada à soropositividade para estas IST (Tabela 1).

Considerando-se a distribuição do total de participantes segundo o IVS, a maioria (n=140; 43,4%) residia em áreas de vulnerabilidade alta/muito alta, 115 (35,6%) em áreas de vulnerabilidade média e 54 (16,7%) em área de vulnerabilidade baixa/muito baixa, além das 14 (4,3%) em situação de rua. A distribuição das participantes com testes reagentes para sífilis, para HIV e para ambos, segundo áreas de vulnerabilidade social, é semelhante àquela descrita para as participantes com TRs não reagentes, não sendo verificada diferença estatisticamente significativa entre as áreas (Tabela 2 e Figura 3).

**Figura 3:** Distribuição espacial das participantes do Projeto TransOdara, com resultados de teste rápido (TR) positivo para sífilis (a), HIV (b) e ambos (c), segundo áreas de vulnerabilidade social, Manaus, 2020-2021

**Tabela 2:** Distribuição em (%) e intervalos de confiança de 95% (IC_95_%) das participantes do Projeto TransOdara, segundo áreas de vulnerabilidade social e resultado do teste rápido (TR) para sífilis e HIV, Manaus, 2020-2021 (n=323)

	TR reagente para sífilis			TR reagente para HIV			TR reagente para sífilis e HIV			Total	
	n	% (IC _95_ % )	p-valor	n	% (IC _95_ % )	p-valor	n	% (IC _95_ % )	p-valor	n	% (IC _95_ % )
**Vulnerabilidade social – área**			0,578			0,885			0,881		
Alta/muito alta	84	42,9 (35,8;50,1)		47	40,5 (31,5;50,0)		36	40,4 (30,2;51,4)		140	43,4 (37,9;48,9)
Média	69	35,2 (28,5;42,3)		43	37,1 (28,3;46,5)		32	36,0 (26,1;46,8)		115	35,6 (30,4;41,1)
Baixa/muito baixa	32	16,3 (11,4;22,3)		21	18,1 (11,6;26,3)		17	19,1 (11,5;28,8)		54	16,7 (12,8;21,2)
Situação de rua	11	5,6 (2,8;9,8)		5	4,3 (1,4;9,8)		4	4,5 (1,2;11,1)		14	4,3 (2,4;7,2)

## DISCUSSÃO 

O presente estudo revelou que a maioria das participantes moravam em áreas classificadas como de *alta/muito alta* e de *média* vulnerabilidade social, onde ainda há carência de oferta de serviços. Juntas, essas regiões concentram cerca de 70% da população de Manaus. A distribuição espacial das participantes do estudo, quando referenciada à população de cada zona, revelou uma maior concentração na zona Sul da cidade e uma baixa presença na zona Centro-Sul, ficando as demais zonas com presença próxima à média do município. 

A zona Centro-Sul é caracterizada por população de alto poder aquisitivo, de classe média alta, com elevado custo de moradia. A zona Sul abriga o centro histórico e comercial, além de serviços de saúde, centros de acolhimento de políticas públicas e de assistência social, voltados à população LGBTQIAP+. A oferta de serviços especializados pode explicar a escolha desta zona da cidade como local de moradia. A região é marcada por fortes contrastes sociais e arquitetônicos, com inúmeras moradias precárias, situadas em áreas vulneráveis, como fundos de vale e margens de igarapés, cursos de água estreitos e pouco profundos. Trata-se, também, de consolidados territórios de prostituição, o que torna a área mais atraente para mulheres trans em situação de vulnerabilidade socioeconômica.^20,21^

O local de moradia não se mostrou associado às taxas de positividade de sífilis e HIV na população do estudo, embora os endereços das participantes fossem distribuídos por todas as áreas analisadas. Esse resultado é semelhante ao de outro estudo realizado no município de São Paulo.^22^

A relação da faixa etária (terem as participantes do estudo mais idade) com os desfechos pode ser explicada pelo maior tempo de exposição a essas IST. Este resultado diverge do obtido em revisão sistemática, com população transexual, considerando os resultados de 25 estudos realizados em 11 países, incluindo o Brasil, onde as maiores prevalências de HIV foram observadas entre as mais jovens.^23^

Também foi associado aos desfechos o baixo nível de escolaridade, indicador clássico de condição de vulnerabilidade social, não só entre a população trans, como também de outros estudos envolvendo populações diversas.^24^ Entretanto, para as MTTr, esta condição pode estar relacionada às altas taxas de evasão escolar, em função da falta de suporte familiar, de experiências de transfobia e da discriminação sofrida.^13,24^

Esta característica pode limitar o acesso à informação necessária de saúde para que elas coloquem em prática estratégias de autocuidado. Além disso, pode dificultar o acesso ao trabalho e renda, e, com isso, o trabalho sexual pode se tornar uma das opções para sua subsistência, o que as deixa mais expostas, em função dos riscos associados a esse tipo de trabalho, como a multiplicidade de parceiros e as práticas sexuais desprotegidas.^14,25^

A proporção de população trans com escolaridade até o ensino fundamental do presente estudo (22%) (TransOdara - Manaus) foi semelhante à observada no TransOdara – município de São Paulo (MSP) (24%),^23^ porém menor do que a verificada no mapeamento da população trans realizado no mesmo município (42%).^26^ Talvez as estratégias de recrutamento adotadas possam explicar as diferenças encontradas no perfil de escolaridade da população trans de cada estudo.

A associação da taxa de positividade dos desfechos com histórico de trabalho sexual do presente estudo (66,4%) foi bem maior do que a apontada em uma revisão sistemática realizada nos Estados Unidos (37,9%); menor que a verificada em estudo de coorte com MTTr, realizado no município do Rio de Janeiro (78,6%);^27^ e semelhante à observada entre as participantes do TransOdara, recrutadas no MSP (68,8%).^22^

Vale mencionar que, embora não tenha sido encontrada associação estatística entre estar em situação de rua, no momento da participação no projeto, e os resultados reagentes de TR para os desfechos analisados, sabe-se que esta é uma condição que vulnerabiliza ainda mais esse grupo, que já enfrenta uma série de dificuldades.^28^ É possível que o número reduzido de participantes nesta condição não tenha permitido evidenciar esta associação. Outra possibilidade é que o risco possa estar mais associado a outras condições socioambientais, características socioeconômicas e comportamentais, ou ainda por essa situação ter sido transitória.

Entre as limitações deste estudo, pode-se elencar o tipo de recrutamento utilizado (RDS), sujeito às características das redes sociais dessa população, que em geral são pouco conhecidas. Dessa forma, recomenda-se cautela na interpretação dos resultados obtidos. É importante reconhecer, entretanto, que, na ausência de melhores estratégias, o RDS continua a ser um método para estudos com populações de MTTr em vários contextos, uma vez que essa é considerada de difícil acesso.^16^ Outra limitação foi a ocorrência da pandemia de covid-19, concomitante ao campo deste estudo, visto que influenciou nas cadeias de recrutamento e na logística dos locais para realização da pesquisa. Isso gerou dificuldades em estabelecer as referências para atendimento a outras demandas de saúde das participantes. Por fim, este estudo, embora não tenha sido originalmente desenhado para analisar a distribuição socioespacial dos desfechos de interesse, contribuiu para evidenciar que estes grupos populacionais estão inseridos, principalmente, em territórios de maior vulnerabilidade social. Vale lembrar ainda que, embora o IVS tenha sido construído com base em dados do Censo de 2010, podendo haver alguma defasagem em relação ao quadro atual, este é o dado disponível, e acredita-se não terem ocorrido alterações mais radicais na distribuição relativa da vulnerabilidade.

Pode-se afirmar que a análise empregada agregou valor, ao indicar os locais prioritários para o direcionamento de políticas públicas que visem promover melhores condições de vida e saúde para esta população.^25,29^ As altas taxas de positividade de sífilis e HIV na população deste estudo e a distribuição preponderante em áreas de vulnerabilidade social alta e muito alta indicam a necessidade de difundir, no território, programas de prevenção, vinculação e retenção às profilaxias e aos tratamentos recomendados.^30^ Isso requer a diversificação de estratégias para ampliar o alcance de populações-chave invisibilizadas, como a de MTTr, e, consequentemente, aumentar a sua adesão aos serviços de saúde.

Em síntese, este estudo contribuiu para caracterizar a distribuição das MTTr participantes do estudo pelos territórios da cidade, além de identificar um subgrupo com vulnerabilidades mais acentuadas, ainda que isso não tenha sido expresso com maior nitidez na distribuição espacial.

Ao ampliar a compreensão dos possíveis mecanismos que levam a essa dinâmica de exclusão e produção de iniquidades a que esta população está submetida, espera-se que este estudo instigue profissionais de saúde, gestores e a sociedade civil a reverterem essa realidade.
